# Raw and processed data used in the simultaneous analysis of electrical characteristics and microstructure of crystallised PEDOT:PSS based OECTs under strain

**DOI:** 10.1016/j.dib.2021.106946

**Published:** 2021-03-15

**Authors:** J.G. Troughton, B. Marchiori, R. Delattre, S. Escoubas, MY. Aliouat, S. Grigorian, M. Ramuz

**Affiliations:** a Department of Flexible Electronics, Mines Saint-Etienne, Center of Microelectronics in Provence, Gardanne, France; b Aix Marseille University, Université de Toulon, CNRS, IM2NP, Marseille, France; c Ecole Nationale Supérieure des Mines et de la Métallurgie, L3M, Annaba, Algeria; d Institute of Physics, University of Siegen, D-57068 Siegen, Germany

**Keywords:** PEDOT:PSS, OECT, GIWASX, Correlating microstructure and performance, SOLEIL, Synchrotron

## Abstract

Here is presented raw and analysed data collected during study of the evolution, with uniaxial stretching, of the electrical and microcrystalline characteristics of polystyrene sulfonate doped poly(3,4-ethylenedioxythiophene) (PEDOT:PSS) organic electrochemical transistors (OECTs). X-ray diffraction data from GIWAXS measurements of the PEDOT:PSS material, performed at the SOLEIL light source are presented in raw and partially analysed forms. Current-voltage data, collected concurrently with the GIWAXS data, are also presented, and the evolution of the transconductance of the OECT devices with stretching is shown. GIWAXS data are only examined along the q_z_ specular reflection ridge, and scans along this ridge are extracted and presented. However, the off-specular data may also be of interest to readers and is therefore made available here in its entirety.

## Specifications Table

**Table:** 

Subject	Materials Physics
Specific subject area	Electrical and materials properties
Type of data	Tables Graph
How data were acquired	Data were acquired using a combination of an X-ray pixel area detector (XPAD) on the DiffAbs beamline of the SOLEIL synchrotron, and a Keithley 2612B source-measure unit. The XPAD and beamline diffractometer were controlled using the Tango Control System. The Keithley was controlled through a custom written LABView program.
Data format	Raw Analyzed
Parameters for data collection	Fabricated PEDOT:PSS OECTs were simultaneously measured with GIWAXS and current-voltage output curves, while being stretched to a progressively greater extent.
Description of data collection	OECTs were fabricated by spin coating PEDOT:PSS on polyurethane. These OECTs were places in the DiffAbs beamline and GIWAXS performed at the same time as output current-voltage sweeps between V_DS_ = 0V and V_DS_ = −0.6 for V_g_ between 0 V and 0.5 V. The strain on the device was increased in variable increments and the measurements repeated until mechanical failure of the device.
Data source location	Institution: Synchrotron SOLEIL City/Town/Region: Saint-Aubin Country: France Latitude: 48.7128003 Longitude: 2.1479529
Data accessibility	Repository name: Mendeley Data Data identification number: http://dx.doi.org/10.17632/v5y3bjw9b6.1 Direct URL to data: http://dx.doi.org/10.17632/v5y3bjw9b6.1
Related research article	J. G. Troughton, B. Marchiori, R. Delattre, S. Escoubas, MY. Aliouat, S. Grigorian, and M. Ramuz, “Simultaneous measurement of electrical characteristics and microstructure of crystallised PEDOT:PSS based OECTs under strain”, Organic Electronics. In Press https://doi.org/10.1016/j.orgel.2021.106108

## Value of the Data

• These data form the basis of the analysis performed in the publication “Simultaneous measurement of electrical characteristics and microstructure of crystallised PEDOT:PSS based OECTs under strain” [1].
• These data are of befit to anyone seeking to better understand the work in the associated publication, as well as anyone interested in verifying the findings.
• These data may be used to directly verify the associated research findings.
• In addition, there is a wealth of further information that may be extracted from the NEXUS raw data files which has not been considered so far, since only the specular (q_z_) diffraction data has been analysed to date. This includes surface morphology and structure correlation lengths, information about which is contained in the off-specular portion of the GIWAXS data.
• As the first known study to simultaneously perform both GIWAXS and electrical measurements on PEDOT:PSS based OECTs, this data will also be of benefit to anyone seeking to replicate and extend this work.

## 1 Data Description

### 1.1 Data contained in the data repository

Dataset 1 contains the Raw NEXUS format data files collected from the DiffAbs beamline while performing GIWAXS measurements. It contains data for the two OECT devices used, along with the same data collected for comparative samples of polyurethane (PU), Parylene C (PaC) coated PU, and glass coated with the PEDOT:PSS mixture with and without acid treatment. Also included are two flat scans performed without any samples in order to correct for non-uniform pixel response in the XPAD, and orientation of the beam, sample, and detector, as described elsewhere [2]. For the two OECT devices tested (Devices A and B), the file name indicates the percentage strain applied during each measurement. An example of a full GIWAXS scan is seen in Fig. 5.

Dataset 2 contains the q_z_ projection of all the GIWAXS measurements contained in Dataset 1, after performing the correction mentioned using the two flat scans. Each GIWAXS dataset is labelled in the same fashion as in dataset 1. These data are used for the fitting and analysis shown in Fig. 6 and in the related paper “Simultaneous measurement of electrical characteristics and microstructure of crystallised PEDOT:PSS based OECTs under strain” [1].

**Fig. 1:**
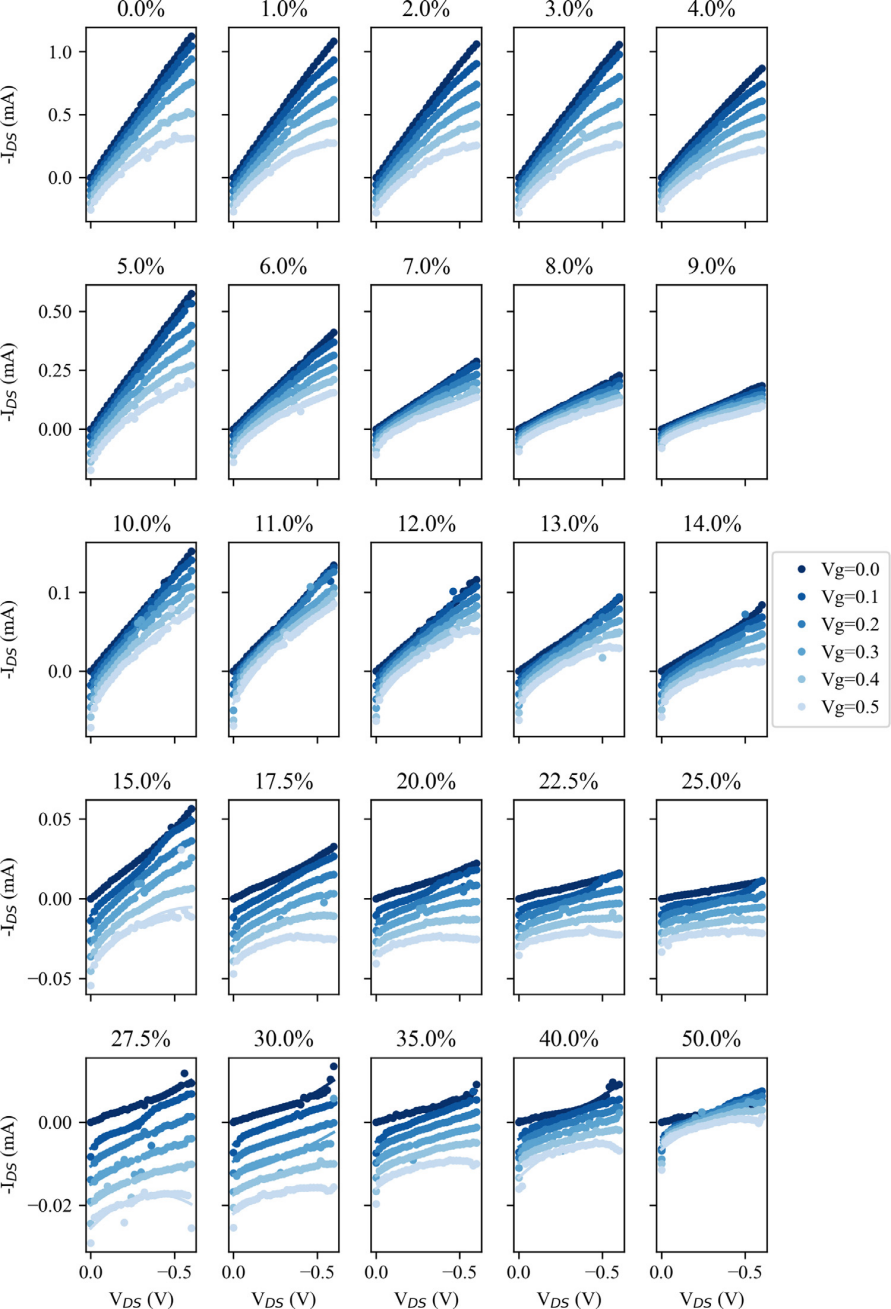
Output curves for Device A at each strain testing point, given as the heading of each subplot. V_g_ is stepped from V_g_=0 V to V_g_ = 0.5 V in 0.1 V intervals as indicated by the colour change. Note that each row has a different I_DS_ scale, which is common across the columns.

**Fig. 2:**
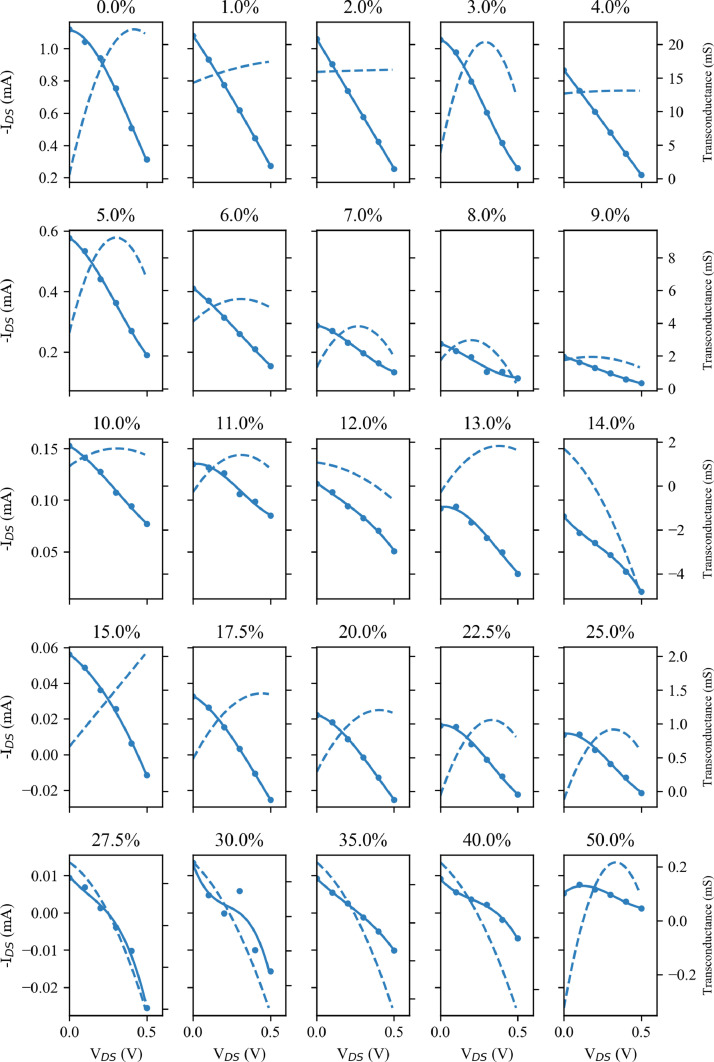
Transfer data for Device A taken from the output curves of Fig. 1 for V_DS_= 0.5 V. The I_DS_ data (solid points) are fitted with a third order polynomial (solid line) and the transconductance taken as the differential of this fitted line (the dashed line). Note that each row has different I_DS_ and transconductance scales, which are common across the columns.

**Fig. 3:**
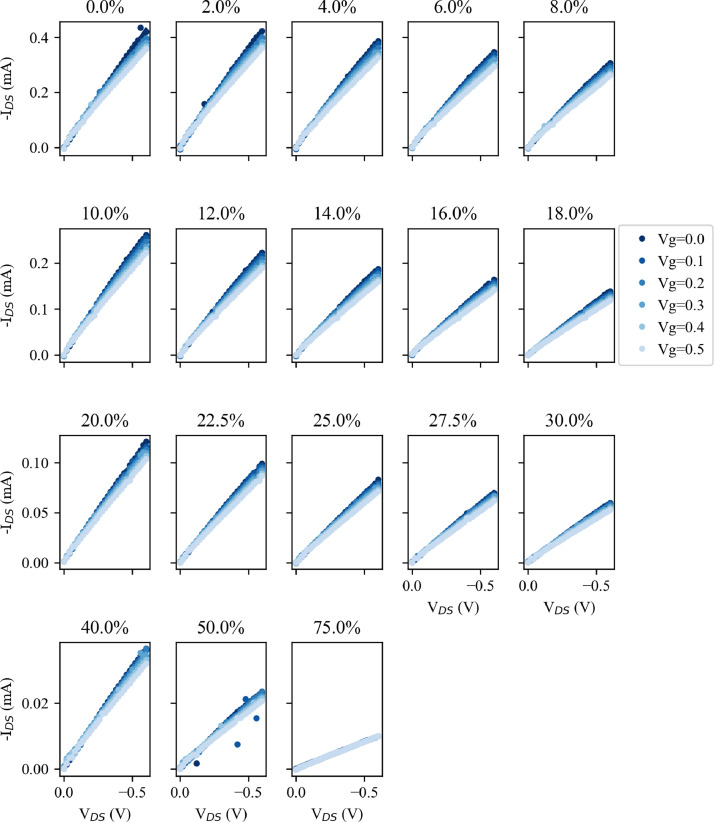
Output curves for Device B at each strain testing point, given as the heading of each subplot. V_g_ is stepped from V_g_=0 V to V_g_ = 0.5 V in 0.1 V intervals as indicated by the colour change. Note that each row has a different I_DS_ scale, which is common across the columns.

**Fig. 4:**
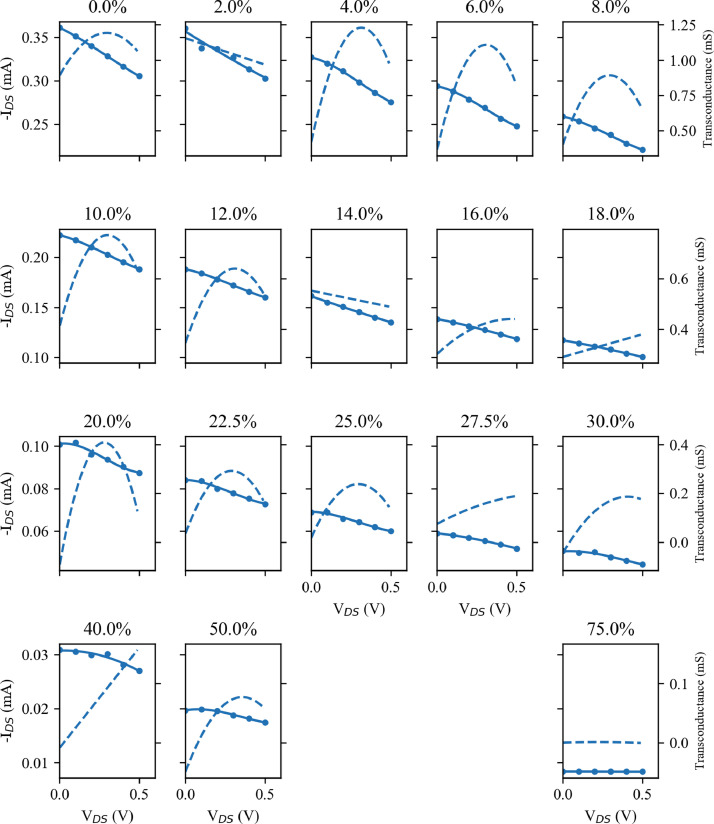
Transfer data for Device B taken from the output curves of Fig. 3 for V_DS_= 0.5 V. The I_DS_ data (solid points) are fitted with a third order polynomial (solid line) and the transconductance taken as the differential of this fitted line (the dashed line). Note that each row has different I_DS_ and transconductance scales, which are common across the columns.

Dataset 3 contains the raw electrical measurements performed on the two OECT devices for each percentage strain. These data form the basis of Figs. 1 to 4 and the electrical data discussed in [1].

### 1.2 Data contained in this article

Fig. 1 shows the output curves (see the following section for a definition of this term and others used in these descriptions) for Device A at each strain testing point. The strain is given as the heading of each subplot. V_g_ is stepped from V_g_ = 0 V to V_g_ = 0.5 V in 0.1 V intervals as indicated by the progressively lighter colour with increasing V_g_. It should be note that each row has a different I_DS_ scale, which is common across the columns. This approach to displaying these data is also used in Figs. 2, 3, and 4. From this, it is clear that the magnitude of I_DS_ decreases with larger strains.

Fig. 2 shows the transfer data for Device A extracted from the output curves of Fig. 1 for V_DS_= 0.5 V. The I_DS_ data are fitted with a third order polynomial and the transconductance taken as the differential of this fitted line. Here, in addition to sharing common I_DS_ scales (displayed on the left of the rows), each row also shares a common transconductance scale (displayed on the right) across the columns. As with the reduction of I_DS_ with strain, there is also a clear fall in the transconductance.

Fig. 3 replicates the display methods of Fig. 1, now showing comparable data for Device B. The falling I_DS_ values are still seen with increasing strain, and this is discussed in the related paper [1].

Fig. 4 replicates the extraction of the transfer data for Device B, and again the falling values of I_DS_ and transconductance can be seen.

**Fig. 5:**
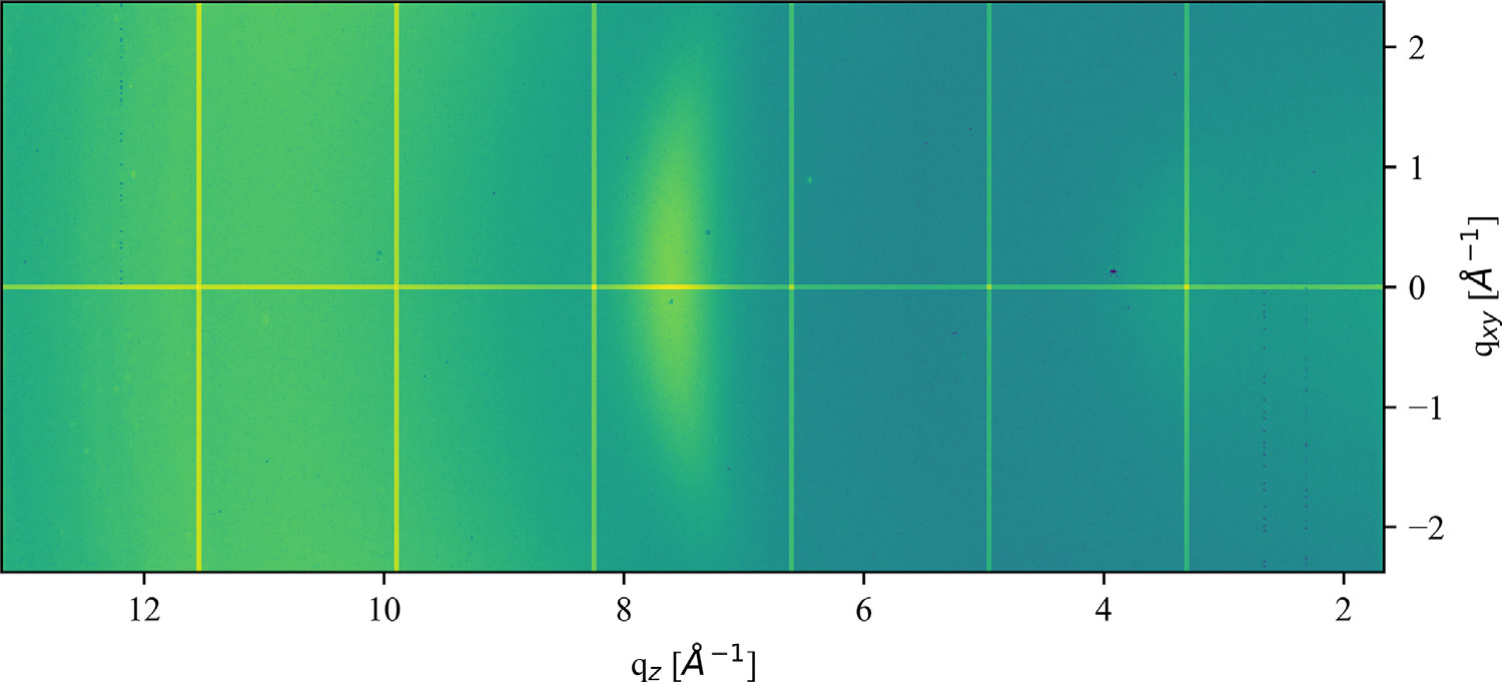
Example full GIWAXS scan taken for Device A before applying any stretching, shown on a log colour scale.

In Fig. 5 an example of a full GIWAXS scan, taken for Device A before applying any stretching, is shown. This is taken from dataset 1 and illustrates the GIWAXS data available. Dataset 2 contains the q_z_ projection of this, which is the q_z_ values along the q_xy_ = 0 Å^−1^, the centre horizontal line through the figure. The gridlines seen are an artefact of the XPAD, and are removed by correcting with the flat scans. This figure can act as a point of reference for future exploration of dataset 1 to ensure treatment is comparable to that discussed here and in the research article [1].

**Fig. 6:**
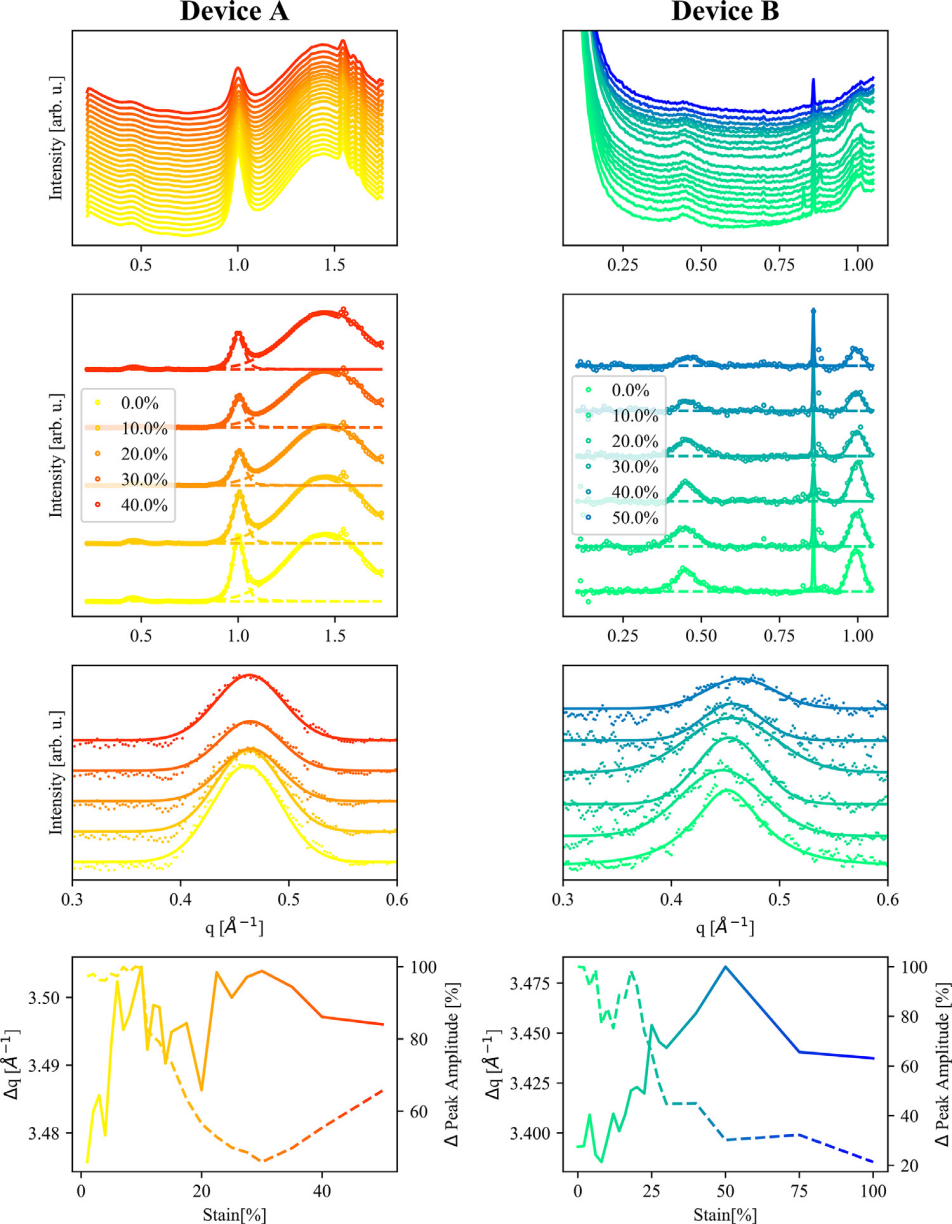
Raw and fitted data from the GIWAXD measurements, taken along the q_z_ direction, for Devices A (left) and B (right). Top Row: Raw data for all measurements coloured following the amount of strain, darker colour indicating more strain. Second Row: A selection of representative curves for each device at different strains (given in the legend) after removal of the background. Data is represented by dots, the fitted peaks are shown with dashed lines, and the total fitted curve is shown with solid lines. For clarity only 1 in 10 data point are shown. Third Row: The same data as the middle panels, enlarged around the peak corresponding to the (100) crystal plane, which forms the focus of study for the associated research article [1]. All data points are now included. Note that all panels in the first three rows include an arbitrary offset in the data in the y-axis to make the figures clearer. Bottom Row: Change in peak position, Δq, and change in peak amplitude, Δ Peak Amplitude, with increasing strain for each device.

Fig. 6 displays raw and fitted data from the GIWAXD measurements in dataset 1 for Devices A and B, taken along the q_z_ direction. These q_z_ projections can be found in dataset 2. At the top is the raw data for all measurements. In the second row is a selection of representative curves for each device at different strains (given in the legend) after removal of the background. These panels also show the peak fitting used for these data (dashed lines), and details of how the background subtraction and peak fitting were performed can be found in the research article [1]. For clarity only 1 in 10 data point are shown. In the third row is the same data as the second row, enlarged around the peak corresponding to the (100) crystal plane, which forms the focus of study for the research article [1]. All data points are now included. Note that all panels in the first three rows include an arbitrary offset in the data in the y-axis to make the figures clearer. The bottom row shows the change in peak position, Δq, and change in peak amplitude, Δ Peak Amplitude, with increasing strain for each device, taken from the peak fitting seen in the third panel.

## 2 Experimental Design, Materials and Methods

### 2.1 Device fabrication

For a detailed description of the device structure and fabrication, see the associated reaserch article [1]. A brief outline of the device structure is provided here for context. The OECT devices comprised a 50 µm polyuthethane (PU) substrate, covered with a 5 µm layer of Parylene C, coated with a 100 nm layer of PEDOT:PSS (Heraeus Clevios PH1000) with the addition of ethylene glycol (Sigma-Aldrich, 0.25 mL for 1 mL PEDOT:PSS solution), and 4,dodecylbenzenesulfonic acid (DBSA) (0.5 µL mL^−1^) which was deposited by spin coating at 1500 rpm for 30 s. A phosphate-buffered saline solution (PBSS) was used as the electrolyte, and a Ag/AgCl probe suspened in the PBSS as the gate electrode.

### 2.2 Grazing insidence wide angle X-ray scattering (GIWAXS)

GIWAXS measurements were performed on the DiffAbs beamline at the SOLEIL light source, using an X-ray pixel area detector (XPAD) described elsewhere [2], with a beam energy of 15 keV, and incidence angle of 0.5°. For Device A an integration time of 3 seconds was used, while for Device B an integration time of 60s was used to compensate for an additional aluminium beamstop with 30 µm pinhole aperture used for beam collimation. The 6+2 circle diffractometer of the DiffAbs beamline, with a XPAD detector, was controlled using the Tango Control System, with the data saved in the NEXUS file format [3] (available in Dataset 1). Further parameter details of each scan can be found in the associated .nxs files found in dataset 1. For help interpreting the .nxs files please see the SOLEIL website [4].

### 2.3 Electrical characterisation

The OECT electrical characterisation was performed using a Keithley 2612B source-measure unit (SMU). For this, the devices were secured in custom fabricated aluminium clamps, which acted as the source/drain contacts of the OECT (see the associated paper for further details). These clamps where connected to one channel of the SMU, and a constant voltage (V_DS_) applied across them while measuring the current flow (I_DS_). A drop of PBSS was applied to one side of the exposed PEDOT:PSS and the gate electrode suspended in the PBSS. This gate electrode was connected to the second channel of the Keithley 2612B to provide the gate voltage (V_g_) while the current flow (I_g_) was also measured. The SMUs were controlled using a custom written LabVIEW script, while the strain applied by the stepper motors was controlled from a second machine using a second LabVIEW program. OECT measurements were performed as a series of seven output sweeps in which V_DS_ was stepped from 0 V to -0.6 V in steps of 0.02 V with a constant V_g_ between 0 V and 0.6 V. In some cases, there is a significant level of current flow from the gate into the channel, contributing to the measured I_DS_. For further analysis this undesired I_g_ was subtracted from the I_DS_, and these measurements, referred to as output curves, can be seen in Figs. 1 and 3. Further parameters can be extracted from these measurements. One alternative way of visualising the performance of these devices is through so-called transfer curves, plotting I_DS_ against V_g_ with a constant V_DS_. Such curves are shown, for V_DS_= 0.5 V in Figs. 2 and 4. From these transfer curves, the device transconductance (a key figure for OECTs) can be calculated. As transconductance is a measure of the change in current, I_DS_, in relation to the change in gate voltage, V_g_, it can be calculated as the derivative of the transfer curve, and this is also shown for V_DS_= 0.5 V in Figs. 2 and 4.

## Ethics Statement

Authors declare that the article is original and unpublished and is not being considered for publication elsewhere, and that it has not been submitted simultaneously anywhere. All authors have checked the revised manuscript and have agreed to the submission. The manuscript has been prepared according to the “Author Guidelines”.

## Declaration of Competing Interest

The authors declare that they have no known competing financial interests or personal relationships which have, or could be perceived to have, influenced the work reported in this article.
